# Molecular Targets of Shenqi Dihuang, A Traditional Chinese Herbal Medicine, and Its Potential Role in Renal Cell Carcinoma Therapy

**DOI:** 10.1155/2023/2355891

**Published:** 2023-01-25

**Authors:** Xinglin Chen, Tongtong Zhang, Xiangyang Zhan, Xinyue Zang, Xinyu Zhai, Zhong Wan, Minyao Ge, Mingyue Tan, Jianyi Gu, Dongliang Xu

**Affiliations:** Urology Centre, Shuguang Hospital Affiliated to Shanghai University of Traditional Chinese Medicine, 528 Zhangheng Road, Pudong New District, Shanghai 201203, China

## Abstract

Chinese herbal medicine (CHM), which includes herbal slices and proprietary products, is widely used in China. Shenqi Dihuang (SQDH) is a traditional Chinese medicine (TCM) formula with ingredients that affect tumor growth. Despite recent advances in prognosis, patients with renal cell carcinoma (RCC) cannot currently receive curative treatment. The present study aimed to explore the potential target genes closely associated with SQDH. The gene expression data for SQDH and RCC were obtained from the TCMSP and TCGA databases. The SQDH-based prognostic prediction model reveals a strong correlation between RCC and SQDH. In addition, the immune cell infiltration analysis indicated that SQDH might be associated with the immune response of RCC patients. Based on this, we successfully built the prognostic prediction model using SQDH-related genes. The results demonstrated that CCND1 and NR3C2 are closely associated with the prognosis of RCC patients. Finally, the pathways enrichment analysis revealed that response to oxidative stress, cyclin binding, programmed cell death, and immune response are the most enriched pathways in CCND1. Furthermore, transcription regulator activity, regulation of cell population proliferation, and cyclin binding are closely associated with the NR3C2.

## 1. Introduction

Renal cell carcinoma (RCC) is the second most lethal tumor of the urinary system's malignant tumors [1]. Clear-cell renal cell carcinoma (ccRCC), papillary renal cell carcinoma (pRCC), and chromophobe renal cell carcinoma (chRCC) are the most common subtypes of RCC [2]. The most common type of RCC in the United States is ccRCC, which accounted for 85% of all cases in 2019 [3]. In addition, approximately 74,000 new cases of ccRCC were diagnosed in 2019. Currently, two major surgical approaches for treating RCC are laparoscopic partial nephrectomy and radical nephrectomy [4]. However, approximately, 30% of patients with ccRCC developed distant metastases that could not be removed surgically. Because ccRCC patients are resistant to radiotherapy, hormones, and cytotoxic treatments [5], several targeted therapies have been approved for metastatic ccRCC, including sunitinib, sorafenib, lenvatinib, and nivolumab [6]. However, the efficacy of these drugs remains limited. Although an increasing number of PD-1/PD-L1 blocking immunotherapy drugs have been approved for the treatment of ccRCC, not all patients respond to them [7]. Therefore, it is clinically significant to determine which patients will benefit from immunotherapy.

Despite recent advances in prognosis over the past decade, patients with metastatic RCC cannot currently receive curative treatment. Cytokine radiation and hormonal therapies have all been studied in combination to reduce relapse rates [8]. Several antiangiogenic medicines, including VEGF pathway inhibitors sunitinib and sorafenib, effectively treat patients with metastatic RCC [9]. Adjuvant sunitinib or sorafenib was superior to placebo in a phase three trial with locally advanced RCC [10].

In recent years, many studies have demonstrated the efficacy of traditional Chinese medicine (TCM) in treating cancer. TCM is widely accepted in China as an effective complementary and alternative therapy for cancer patients [11]. Chinese medicine has been used throughout Asia since ancient times. The most common application category of TCM is Chinese herbal medicine (CHM), which includes herbal slices and proprietary products [12]. Because CHM is effective and has fewer side effects, it is used as an alternative therapy by many cancer patients [13]. Shenqi Dihuang (SQDH) is a TCM formula containing ingredients that inhibit tumor growth [14]. Ginseng, *Astragalus membranaceus*, rehmannia, yam, tuckahoe, paeonol, and dogwood are among the ingredients in SQDH. There is evidence that the traditional Chinese herbal formula has fewer side effects and is more cost-effective than other treatments [15]. Previous studies demonstrated that their effects are mediated by immune cell activation and reprogramming metabolic-related inflammatory responses [16].

With the development of bioinformatics analysis, many researchers have started exploring the potential prognostic factors for multiple tumors. The present study aimed to investigate the potential correlation between SQDH and RCC. In addition, immune infiltration analysis was used to reveal the relationship between the immune response of RCC patients and SQDH. Furthermore, the prognostic prediction model was developed to investigate the genes closely associated with the prognosis of RCC patients. Finally, the pathway enrichment analysis was performed to explore the potential pathways closely linked to the SQDH. Our study aims to investigate the role of SQDH in RCC immunotherapy.

## 2. Methods

### 2.1. Datasets Downloaded

Traditional Chinese Medicine System Pharmacology Database and Analysis Platform (TCMSP) (http://tcmspw.com/tcmsp.php) was used to obtain SQDH composition and molecular target data. Furthermore, the expression data and clinical characteristics of RCC patients were downloaded from The Cancer Genome Atlas (TCGA) database.

### 2.2. Differentially Expressed Analysis

The Cancer Genome Atlas (TCGA) database (https://portal.gdc.com) was used to obtain RNAseq data and associated clinical information. The Limma R software package was used to investigate mRNA expression differences. A threshold differential expression screen for mRNA was defined as “*P* < 0.05 and log 2 (fold change) > 2 or log 2 (fold change) < −2.”

### 2.3. Functional Analysis Based on Gene Ontology (GO) and Kyoto Encyclopedia of Genes and Genomes (KEGG) Enrichment Pathways

The data were analyzed using feature enrichment to confirm the possible functions of potential targets. Using GO, it is a common practice to annotate genes with functions, particularly molecular function (MF), biological pathway (BP), and cellular component (CC). An enrichment analysis based on KEGG can be used to investigate gene function and related high-level genomic information. To better understand the oncogenic role of target genes, ClusterProfiler in R was used to analyze the GO functions and KEGG pathway.

### 2.4. Protein-Protein Network (PPI) Analysis Based on SQDH-Related Genes

The PPI network was then constructed to investigate the potential correlation between the proteins encoded by key genes. STRING was used to perform an interactive analysis of a gene PPI network. Furthermore, Cytoscape 3.7.2 was used to analyze and visualize PPI networks when interactions with composite ratings exceeded 0.9.

### 2.5. Immune Cell Infiltration

To investigate the correlation between the built MRGS and immune cell infiltration, we estimated the infiltration levels of 22 immune cell subtypes in the RCC cohort using CIBERSORT. Enrichment scores calculated by ssGSEA of R's Gene Set Variation Analysis package were used to quantify immune cell infiltration. This analysis revealed information about immune infiltration, such as immune cell species, immune functions, and immune-related pathways.

### 2.6. Construction of the Prognostic Prediction Model Based on the SQDH Target Genes

The prognostic prediction model was built using univariate and multivariate COX regression analyses. In addition, the survival analysis was used to compare the overall survival (OS) of RCC patients in low- and high-risk groups. Furthermore, an area under the receiver curve (AUC) was determined using the receiver operating characteristic curve (ROC).

### 2.7. Statistical Analysis

Statistical analysis was performed using R software. The difference between groups was statistically significant, with a *P* value <0.05.

## 3. Results

### 3.1. The Potential SQDH Target Genes Pathways and the Protein-Protein Network Based on SQDH-Related Proteins

**Figure 1:**
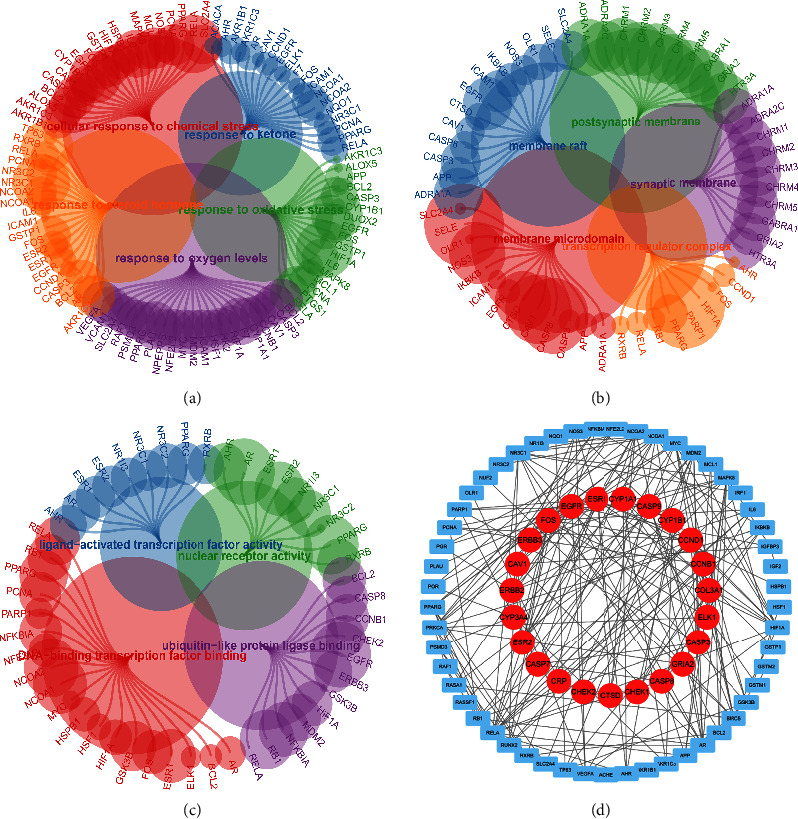
(a) GO BP enrichment analysis based on the SQDH-related genes. (b) GO CC enrichment analysis based on the SQDH-related genes. (c) GO MF enrichment analysis based on the SQDH-related genes. (d) PPI network based on the SQDH-related genes.

Based on the ingredients in SQDH, *Codonopsis pilosula*, *Poria cocos*, and *Astragalus membranaceus* were considered the most important ingredients. Subsequently, the TCMSP database was used to obtain the target genes of *Codonopsis pilosula*, *Poria cocos*, and *Astragalus membranaceus*. A total of 108 genes were identified as SQDH target genes. The GO and KEGG enrichment analyses were performed to investigate potential pathways closely associated with SQDH. The results demonstrated that most GO BP pathways are cellular responses to chemical stress, ketone, a steroid hormone, oxidative stress, and oxygen levels (Figure 1(a)). Regarding CC, membrane raft, postsynaptic membrane, membrane microdomain, synaptic membrane, and transcription regulator complex are closely associated with SQDH-related genes (Figure 1(b)). In addition, the GO MF enrichment analysis revealed that the most enriched pathways involved in SQDH-related genes are ligand-activated transcription factor activity, nuclear receptor activity, DNA-binding transcription factor binding, and ubiquitin-like protein ligase binding (Figure 1(c)). The results of the PPI network revealed that 81 SQDH-related genes were closely related to one another. Furthermore, some genes, known as hub genes, had more than 20 interactive counts with other genes, including ESR1, RELA, FOS, AR, CCND1, NCOA1, MAPK8, EGFR, HIF1A, NR3C1, MDM2, and PRKCA (Figure 1(d)).

### 3.2. Exploration of the Differentially Expressed Genes between RCC Patients and Normal People

**Figure 2:**
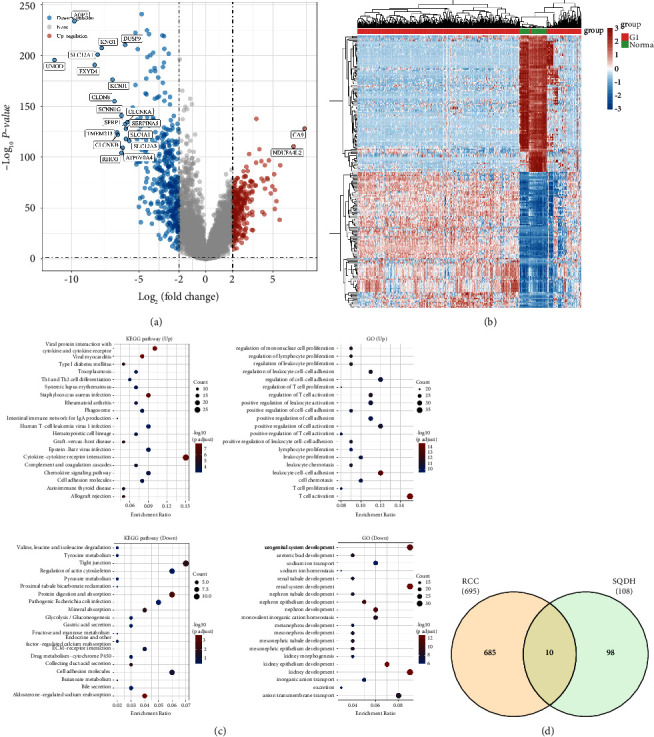
(a) The differential expression analysis based on the RCC cohort in the TCGA database. (b) The heat map demonstrated the differentially expressed genes between RCC and normal tissue. (c) The GO and KEGG enrichment analysis based on differentially expressed genes. (d) The Venn diagram displayed the genes that are closely associated with RCC and SQDH.

A total of 532 RCC patients and 72 normal people were included in the TCGA cohort. The fold change was set into 2 to explore the genes closely associated with RCC. The differentially expressed analysis revealed 695 differentially expressed genes, including 278 upregulated and 417 downregulated genes (Figures 2(a)–2(b)). The pathways enrichment analysis demonstrated that some immune-related pathways, such as regulation of T cell activation, regulation of T cell proliferation, and Th1 and Th2 cell differentiation, are closely linked to the differentially expressed genes. In addition, the most enriched pathways are renal tubule development, renal system development, kidney morphogenesis, and kidney epithelium development. The target genes and active ingredients of *Codonopsis pilosula*, *Astragalus membranaceus*, and *Poria cocos* were then obtained from the TCMSP dataset. Finally, 108 target genes associated with *Codonopsis pilosula*, *Astragalus membranaceus*, and *Poria cocos* were downloaded (Figure 1(c)). The Venn diagram demonstrated that ten genes, including HK2, VEGFA, IGFBP3, CAV1, ALOX5, CCND1, DIO1, NR3C2, ADH1B and PTGER3, are closely related to the differentially expressed genes in the RCC cohort and SQDH targets genes (Figure 2(d)).

### 3.3. Construction of the SQDH-Related Prognostic Prediction Model

**Figure 3:**
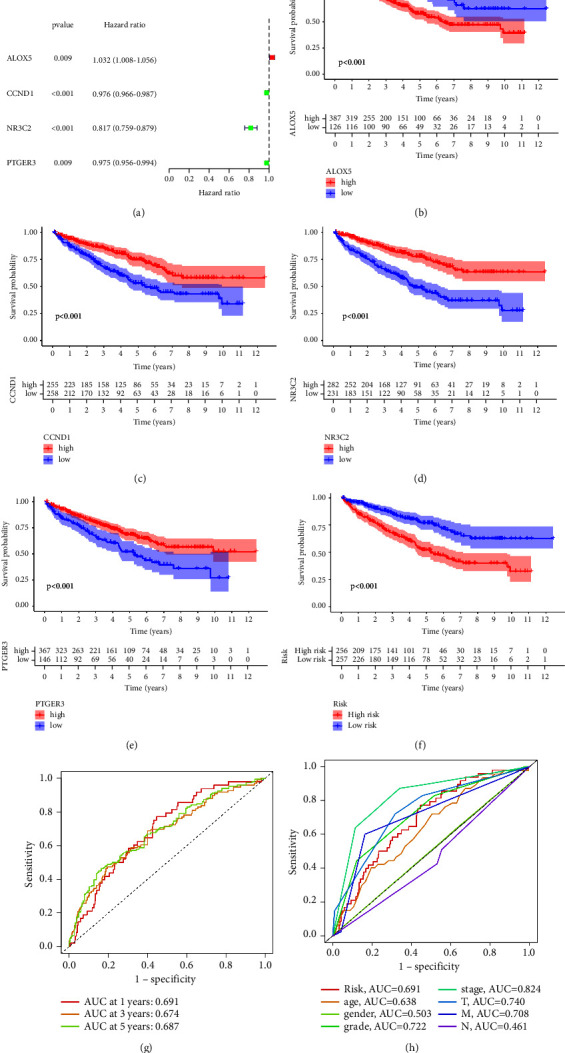
(a) The univariate COX regression analysis based on the OS of RCC patients. (b) Survival analysis between low- and high-expression of ALOX5 groups. (c) The survival analysis between low- and high-expression of CCND1 groups. (d) The survival analysis between low- and high-expression of NR3C2 groups. (e) The survival analysis between low- and high-expression of PTGER3 groups. (f) The survival analysis between low- and high-risk groups. (g) The clinical-related ROC curve demonstrated the predictive value of clinical characteristics and risk score. (h) The time-dependent ROC curve demonstrated the predictive value of prognostic prediction model.

Based on the previous analysis, ten genes were thought to be closely related to the prognosis of RCC patients. The expression matrix of RCC patients was obtained by combining the expression data and the clinical information of RCC patients. Subsequently, the univariate COX regression analysis reveals that ALOX5, CCND1, NR3C2, and PTGER3 are strongly linked to the prognosis of RCC patients (Figure 3(a)). The multivariate COX regression analysis revealed that the prognostic prediction model was built using CCND1 and NR3C2. The risk score is: risk score = −0.0197007190065525 ∗ CCND1 + −0.167437463401867 ∗ NR3C2. Then, we performed a survival analysis based on the expression level of ALOX5, CCND1, NR3C2, and PTGER3. The results demonstrated that the high-expression levels of CCND1, NR3C2, and PTGER3 are associated with a better OS in RCC patients. However, the high ALOX5 expression is associated with poorer OS in RCC patients (Figures 3(b)–3(e)). In addition, the survival analysis based on the risk score revealed that RCC patients in the high-risk group have a poorer OS (Figure 3(f)). Finally, we performed the ROC curve. The results demonstrated that the 1-year, 3-year, and 5-year AUC are >0.6, indicating that the model has a good predictive value (Figure 3(g)). Furthermore, the clinical-related ROC curve demonstrated that the prognostic prediction model and clinical characteristics could be used as predictive factors (Figure 3(h)).

### 3.4. The SQDH-Based Prognostic Prediction Model Is Closely Associated with Many Immune Cells

**Figure 4:**
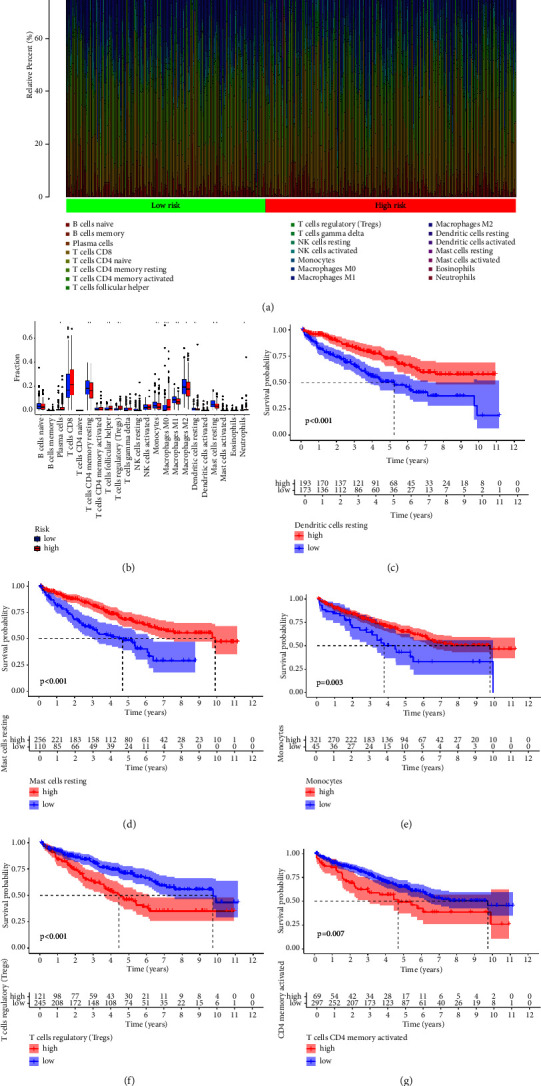
(a)–(b) The difference in immune cells between low- and high-risk groups. (c) Survival analysis based on the resting T cells expression level. (d) Survival analysis based on the resting mast cells expression level. (e) Survival analysis based on the expression monocytes level. (f) Survival analysis based on the regulatory T cells expression level. (g) Survival analysis based on the activated T cells expression level.

The immune cell infiltration analysis was then performed using the SQDH-related prognostic prediction model. Some immune cells were closely associated with the risk score, including plasma cells, CD8 T cells, CD4 memory resting T cells, follicular helper T cell, regulatory T cell, monocyte, and M0, M1, and M2 macrophages (Figures 4(a) and 4(b)). In addition, the distribution of some immune cells is linked to the OS of RCC patients. The results demonstrated that the RCC tissues with high resting dendritic cells, resting mast cells, and monocytes have a better OS. However, the higher number of T regulatory cells and activated memory CD4 T cells is associated with worse OS (Figures 4(c)–4(g)).

### 3.5. Some Immune-Related Functions Are Closely Associated with the SQDH-Based Prognostic Prediction Model

**Figure 5:**
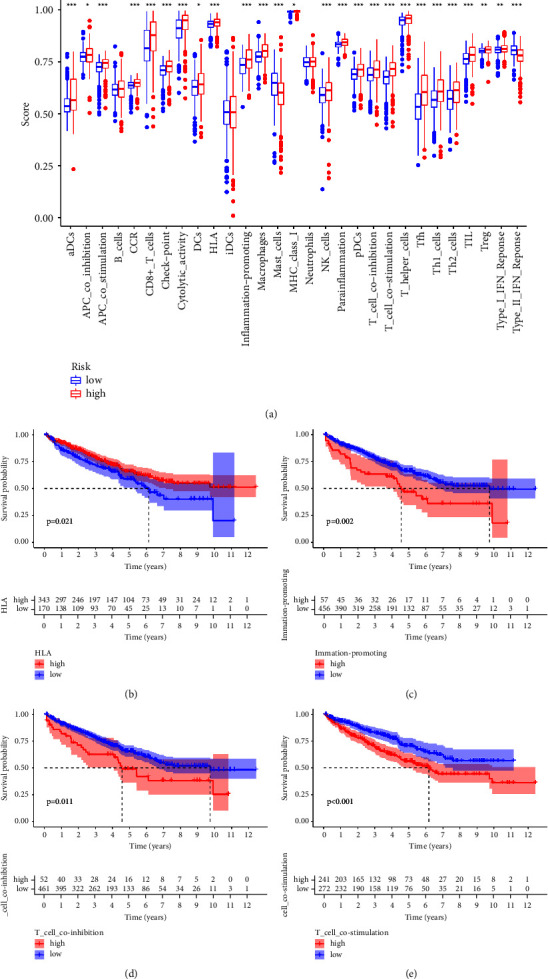
(a) The correlation between SQDH-based prognostic prediction model of immune-related functions. (b) The survival analysis between low- and high-HLA groups. (c) The survival analysis between low- and high-inflammation-promoting groups. (d) The survival analysis between low- and high-co-inhibition T cell groups. (e) The survival analysis between low- and high-co-stimulation T cell groups.

We then compared immune-related function between low- and high-risk groups using the risk score for RCC patients and immune cell infiltration analysis. Some immune-related functions, such as aDCs, immune checkpoint, human leukocyte antigen (HLA), type I and type II IFN responses, T cell co-stimulation and co-inhibition factors, are found to be significantly different (Figure 5(a)). In addition, some immune-related functions are linked to the OS of RCC patients. RCC patients with higher HLA have a better OS. However, the higher levels of inflammation-promoting factors and T cell co-inhibition and co-stimulation factors are correlated with a poorer OS in RCC patients (Figures 5(b)–5(e)).

### 3.6. CCND1 and NR3C2 Were Closely Associated with Many Enriched Pathways Involved in RCC Patients

**Figure 6:**
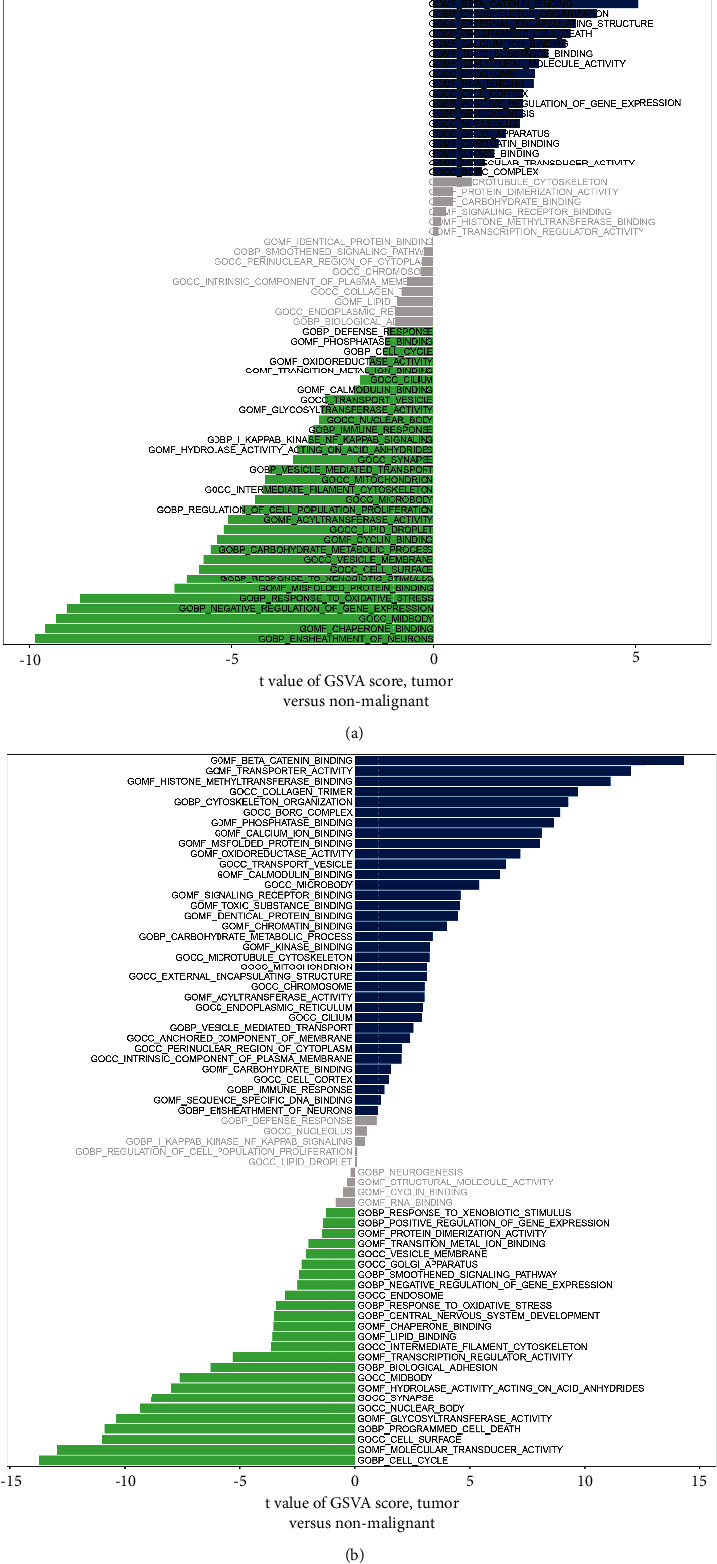
(a) The GSVA analysis of low- and high-CCND1 groups. (b) The GSVA analysis of low- and high-NR3C2 groups.

CCND1 and NR3C2 build the SQDH-based prognostic prediction model. Subsequently, we aimed to explore the potential pathways closely linked to CCND1 and NR3C2. The most enriched pathways for CCND1 are a response to oxidative stress, central nervous system development, carbohydrate metabolic process, regulation of cell population proliferation, cyclin binding, programmed cell death, and immune response (Figure 6(a)). In addition, transporter activity, beta-catenin binding, transcription regulator activity, identical protein binding, regulation of cell population proliferation, and cyclin binding are all closely associated with the NR3C2 expression (Figure 6(b)).

## 4. Discussion

RCC is the sixth most common malignancy in men and the tenth most common in women, accounting for 5% and 3% of all cancers, respectively [17]. The incidence of RCC has increased over time. Although surgery remains the primary treatment option for patients with locally or locally advanced disease, a significant proportion of patients will eventually experience disease recurrence [18]. Chemo- and radiotherapy are ineffective in treating RCC. Immunotherapy has recently been implemented due to a better understanding of RCC biology [19]. The antitumor activity of sunitinib can be attributed to its multichannel nature as a tyrosine kinase inhibitor [20]. A phase II study conducted independently in two separate groups revealed that sunitinib significantly delayed tumor progression and had a high treatment response rate. The ORR in both trials was 42%, with a median time to disease progression (TTP) of 8.7 months [21].

Furthermore, sunitinib was more effective in phase III clinical study of patients with metastatic RCC than IFN-*α* [22]. It is also likely to cause serious side effects such as nausea, vomiting, diarrhea, rash, hand-foot syndrome, and others that will significantly impair the patient's ability to adhere to their treatment and live a good life [23]. TCM treatment is also used to reduce the side effects of targeted drug therapy and promote recovery of patients' body function in advanced cancer patients [24]. In addition, analysis of the online dataset has been widely applied in the various of human diseases [25–27].

SQDH consists of ginseng, *Astragalus membranaceus*, rehmannia, yam, tuckahoe, paeonol, and dogwood. The SQDH boosts the body's humoral and cellular immunity, accelerates tumor cell apoptosis, inhibits angiogenesis, regulates cytokines, and slows metastasis [28]. Polysaccharides have immune-regulatory and antitumor properties in *Codonopsis pilosula* [29]. A variety of polysaccharides, saponins, and other compounds found in *Astragalus membranaceus* can regulate tumor immunity, influence tumor cell autophagy, and inhibit tumor angiogenesis [30]. The present study aimed to explore the association between RCC and SQDH using a network pharmacology approach.

The main ingredients and target genes of SQDH were obtained from the TCMSP database, and relative pathways closely associated with the development and progression of ccRCC were identified. The PPI network based on the SQDH-related genes also revealed that these genes are highly correlated. In addition, the TCGA database was searched for RCC differentially expressed genes. The Venn diagram was then used to investigate the genes linked to RCC and SQDH. The SQDH-based prognostic prediction model reveals that two SQDH target genes (CCND1 and NR3C2) were closely associated with RCC patient prognosis. The survival analysis and the ROC curve demonstrated that the model has an excellent predictive value for RCC patients. Finally, we investigate the role of the SQDH-based model in the immune response of RCC patients. The findings revealed that certain immune checkpoints, immune cells, and functions are highly correlated with the risk model. Our results indicated that CD4+ T cells and macrophages are closely linked to the SQDH-based prognostic prediction model. In addition, the HLA and immune checkpoint are also closely associated with the model, implying that SQDH may play an important role in the immunotherapy of RCC patients. Further investigation revealed that CCND1 and NR3C2 are the key SQDH-target genes closely related to the RCC.

In conclusion, we identified the close relationship between SQDH and RCC. In addition, the SQDH-based prognostic prediction model reveals that SQDH may influence the immune response of RCC patients. In addition, two key genes (CCND1 and NR3C2) may play an important role in the immunotherapy process for SQDH and RCC patients. By further investigating its mechanism, it may be possible to develop a basis for combining TCM and advanced renal cancer.

## Data Availability

The data used to support the findings of this study are included within the article.
